# Stercoral Ulcer Not Always Indolent: A Rare Complication of Fecal Impaction

**DOI:** 10.7759/cureus.2613

**Published:** 2018-05-13

**Authors:** Chellappa Vijayakumar, K Balagurunathan, Ravi Prabhu, Erabati Santosh Raja, Singh Amankumar, Raja Kalaiarasi, Swetha T

**Affiliations:** 1 Surgery, Jawaharlal Institute of Postgraduate Medical Education and Research (JIPMER), Puducherry, India.; 2 Surgery, Sri Lakshmi Narayana Institute of Medical Science, Puducherry, India; 3 General Surgery, Sri Lakshmi Narayana Institute of Medical Science, Puducherry, India; 4 Otorhinolaryngology, Sri Lakshmi Narayana Institute of Medical Science, Puducherry, India; 5 Obstetric and Gynaecology, Mahatma Gandhi Medical College and Research Institute, Pondicherry, India

**Keywords:** stercoral, fecal peritonitis, sepsis, constipation, colonic perforation

## Abstract

Stercoral sigmoid perforation is a dangerous surgical emergency. It is also a life-threatening situation because the spillage of fecal contents into the abdominal cavity leads to sepsis with many postoperative complications. Chronic, intermittent constipation can lead to fecal impaction, especially in older patients.

An 80-year-old male patient presented with intestinal abdominal pain and distention for three days. His chest X-ray showed air under the diaphragm. On laparotomy, a small rent was discovered in the rectosigmoid junction with fecal contamination. The presence of a fecaloma is the speculated reason for the perforation. Primary closure of the defect with a diverting transverse colostomy was performed, and subsequently, the patient recovered well. A colostomy closure was performed six weeks after the primary surgery.

It is imperative to understand the incidence of stercoral perforation in a normal bowel. Early treatment and intervention are the important aspects of stercoral pathology. We report a rare case of stercoral sigmoid colonic perforation with fecal peritonitis.

## Introduction

Fecal impaction is one of the common causes of subacute intestinal obstruction in elderly patients [1]. It is mostly due to the impaction of a hard fecal mass which occludes the intestine at any level. The most common site of obstruction is the rectosigmoid junction. These impacted stools cannot be evacuated spontaneously or manually. On rectal examination, 7% of patients are found to have impacted stools [2]. They are prone to hollow viscus perforation. The majority of the cases are a gastric perforation, ileal perforation in typhoid fever, and post-traumatic small bowel perforation. Large intestinal perforations are very rare, but it usually occurs in patients with inflammatory bowel diseases, diverticular anomalies, and malignancies.

Stercoral perforation is due to the hard impacted stool which affects the normal defecation process. Any delay in diagnosis and treatment could result in catastrophic consequences. The hard fecal mass may compress the ureter causing hydronephrosis or the vascular structures, resulting in limb ischemia [3]. Therefore, it is vital to treat this type of intestinal obstruction as early as possible to prevent mortality (35%).

Perforation followed by fecal contamination in peritoneal cavity leads to sepsis. Diverticular anomalies and malignancies are the common causes for stercoral peritonitis. Sepsis due to fecal contamination has a high morbidity and mortality rate. Perforation preceded by an ischemic colonic wall usually occurs due to high colonic pressure. Sometimes these conditions activate inflammatory sequences which lead to stercoral colitis [4]. The most common site for stercoral perforation is the sigmoid region, especially in the rectosigmoid junction. The purpose of this case report is to identify stercoral perforation at an early stage since the mortality rate can be substantially reduced by early intervention of the stercoral perforation.

## Case presentation

**Figure 1:**
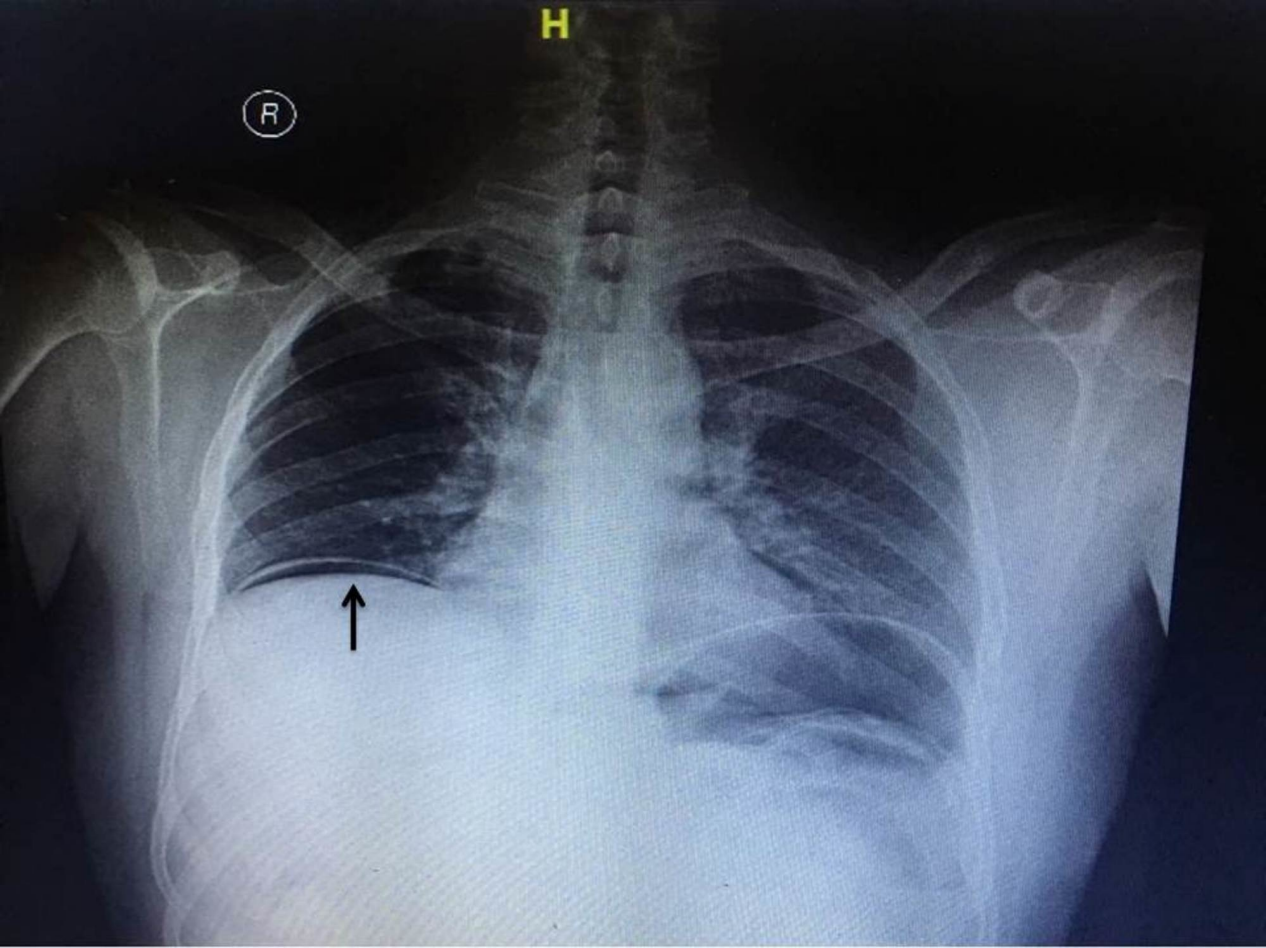
Preoperative x-ray with air under diaphragm (black arrow)

An 80-year-old male patient was admitted to the emergency ward with the complaints of abdominal distention, vomiting, and constipation for three days. On examination, the patient was dehydrated with stable vital signs. The abdomen was distended with generalised guarding and rigidity. The bowel sounds were not heard. On rectal examination, rectum was empty with minimal fecal staining and no palpable mass lesion. After initial resuscitation, imaging studies, including an ultrasound of the abdomen, were done. Chest X-ray showed air under the diaphragm (Figure 1).

**Figure 2:**
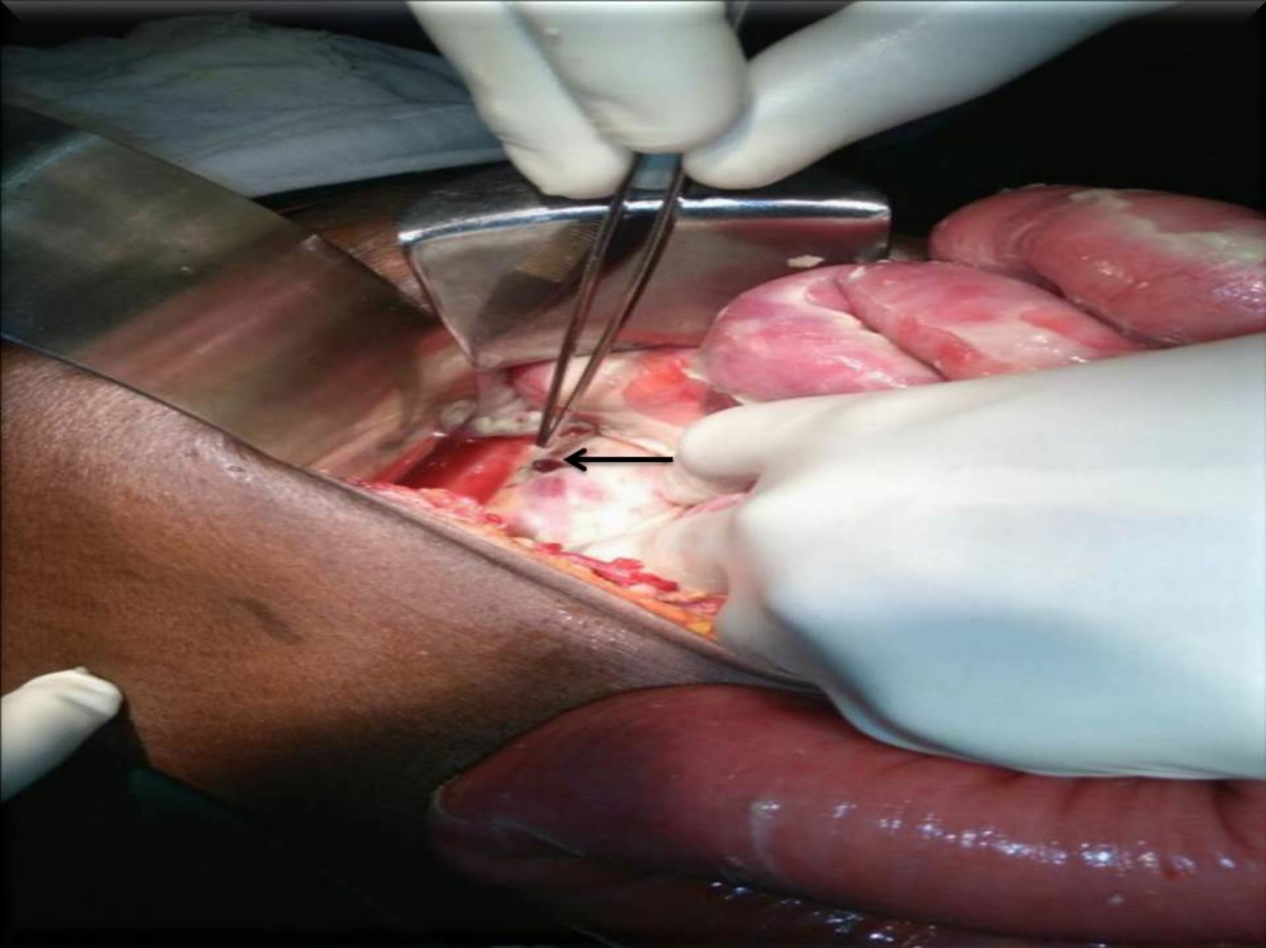
Intraoperative sigmoid perforation (black arrow)

Abdominal x-ray showed few air-fluid levels. Ultrasound showed distended bowel loops with significant free fluid in the peritoneal cavity. The patient was diagnosed to have a hollow viscus perforation with peritonitis. Routine blood investigations and blood cultures were done. Renal parameters were deranged suggesting pre-renal failure. Broad spectrum antibiotics were started in view of the high leukocyte counts. The patient was taken up for emergency laparotomy. Intraoperatively, approximately 500 mL fecal-contaminated peritoneal fluid was cleared. There was a 1 x 1 cm^2^ rent in the rectosigmoid junction (Figure 2).

Proximal to the perforation, large bowel loops were distended. Bowel wall thickening was present in the rectosigmoid junction. Distal to the perforation, the rectum was found to be collapsed. The liver and the rest of the intestine were normal. Pelvic deposits/growths were not found. Primary closure of the rent in two layers was done after a thorough peritoneal wash. A diverting transverse colostomy was done after manual bowel decompression. The patient recovered after one week of intensive postoperative care. He was started on oral fluids on the tenth postoperative day and discharged after three weeks of hospital stay. The patient was followed up regularly, and the colostomy closure was done six weeks after the primary surgery. 

## Discussion

Hollow viscus perforation is the most common cause of peritonitis and pneumoperitoneum. Perforation due to hard impacted stools is rare. Very few cases have been reported in the literature. Stercoral perforation was first identified by Berry in 1894 [5]. Perforation usually occurs due to pressure necrosis in a diseased bowel. Stercoral perforation should be diagnosed in the absence of any other bowel pathology. Hard impacted stools are formed because of poor hydration of the stools. Usually, patients have altered bowel habits for a long duration. In this case, poor dietary fibre and less water intake resulted in chronic constipation and stercoral perforation. This partly explains the increased incidence in developing countries [1].

Hard stool cause colonic wall ulceration (stercoral ulcer) which leads to stercoral perforation. Instead of pressure necrosis, ischemic necrosis also plays a role in ischemic colitis. The most common site of stercoral perforation is the rectosigmoid junction [6]. Perforation usually occurs in the antimesenteric border, especially below the peritoneal reflection, due to early vascular compromise in the antimesenteric border. Also, the intraluminal pressure is high in the retroperitoneal region compared to the intraperitoneal cavity. Narrower luminal diameter in the rectosigmoid junction causes hard stools to be stagnant at the junction. Untreated constipation aggravates the intraluminal pressure further. This explains the need for early treatment to avoid colonic ischemia.

Old age, frequent intake of NSAIDS (non-steroidal anti-inflammatory drugs), and intermittent constipation are common risk factors for stercoral pathology [7]. Stercoral perforation is also common in younger individuals suffering from neurological problems [8]. High-risk patients should be advised to increase water intake, along with a high fiber diet, fruits, and stool softeners. A chest x-ray is mandatory for all cases of hollow viscus perforation cases. Though plain abdominal x-rays can diagnose fecaloma with ease in advanced diseases, computerised tomography (CT) is the investigation of choice. Pericolonic fat stranding is the characteristic feature of colonic ischemia [9]. There are few diagnostic criteria described in the literature. The perforation should be oval and more than 1 cm in diameter in the antimesenteric border of the colon. The hard fecal matter should extrude from the perforation. Fecal contamination, or sometimes a fecaloma, should be present in the peritoneal cavity and the perforated bowel should be free from any pathology. In the histopathological report, chronic inflammatory changes should be identified by microscopy [9].

Stercoral pathology should be suspected in elderly patients with altered bowel habits, especially chronic constipation. Stercoral perforation is a diagnosis of exclusion, and its diagnosis should be ruled out when another bowel pathology coexists. Chronic constipation may be due to drug-induced or neurological problems. More often, laboratory parameters may not diagnose the underlying pathology. Abdominal x-rays are rarely useful to diagnose fecaloma. A CT scan is the ideal investigation for suspected stercoral pathology. An adequate knowledge about stercoral pathology can significantly reduce the mortality rate (35%) [10]. Since it is a surgical emergency, early diagnosis and treatment can be life-saving.  

## Conclusions

In elderly patients with a chronic history of severe constipation presenting with symptoms of acute intestinal obstruction, the possibility of stercoral perforation should be considered. A preoperative CT scan is the ideal investigation to diagnose stercoral pathology. Early diagnosis and early intervention can significantly reduce mortality due to fecal peritonitis.
